# Evaluating disability, comorbidities and risk factors after TB treatment: an 18–24 month follow-up

**DOI:** 10.5588/ijtldopen.25.0149

**Published:** 2025-05-12

**Authors:** Y. Sun, Y. Lin, J.E. Golub, W. Shu, J. Jiang, Q. Xu, Y. Li, W. Sun, Y. Shi, J. Liao, C. Nie, C. Liang, X. Zhang, H. Liu, Y. Ma, R. Zachariah, S.D. Berger, P. Thekkur, D. Nair, S. Satyanarayana, A.M.V. Kumar, A.D. Harries

**Affiliations:** ^1^ Clinical Center on TB, Beijing Chest Hospital, Capital Medical University, Beijing 101149, China;; ^2^ Beijing Tuberculosis and Thoracic Tumor Research Institute, Beijing 101149, China;; ^3^ International Union Against Tuberculosis and Lung Disease, Paris, France;; ^4^ International Union Against Tuberculosis and Lung Disease, Beijing, China;; ^5^ Johns Hopkins Centre for Tuberculosis, Johns Hopkins University School of Medicine, Baltimore, USA;; ^6^ The Third People's Hospital of Yichang City, Yichang, Hubei 443000, China;; ^7^ Wuhan Pulmonary Hospital, Wuhan, Hubei 430030, China;; ^8^ The First People’s Hospital of Yongkang, Jinhua, Zhejiang 321300, China;; ^9^ Ezhou Third Hospital, Ezhou, Hubei 436000, China;; ^10^ Dongyang People’s Hospital, Dongyang, Zhejiang 322100, China;; ^11^ The People's Hospital of Laibin, Laibin, Guangxi 546100, China;; ^12^ Xiangyang Institute of Tuberculosis Control and Prevention, Xiangyang, Hubei 441000;; ^13^ Baise City People’s Hospital, Baise, Guangxi 533000, China;; ^14^ Zhongwei People's Hospital, Zhongwei, Ningxia 755000, China;; ^15^ Guangxi Chest Hospital, Liuzhou, Guangxi 545000, China;; ^16^ The People's Hospital of Tongxin, Wuzhong, Ningxia 751100, China;; ^17^ TDR, Special Programme for Research and Training in Tropical Diseases (TDR), Geneva, Switzerland;; ^18^ The Union South-East Asia Office, C-6 Qutub Institutional Area, New Delhi, India;; ^19^ Yenepoya Medical College, Yenepoya (Deemed to be University), Mangalore, India;; ^20^ London School of Hygiene and Tropical Medicine, London, UK.

**Keywords:** tuberculosis, China, health systems, multimorbidity, employment status, real-time operational research

## Abstract

**BACKGROUND:**

Several countries have developed national strategic plans to address post-TB disability and comorbidities. However, their feasibility and added value within routine programmatic settings remain undocumented.

**METHODS:**

We followed up individuals who successfully completed TB treatment at 11 health facilities in China between 2022–2023. Within the programmatic setting, we assessed health status, on-going symptoms, comorbidities, risk factors and disability (measured by 6-minute walking test [6MWT]) 18–24 months after treatment completion.

**RESULTS:**

Of 586 individuals who completed TB treatment, 503 (86%) were reassessed. Compared with end of TB treatment, there were significant increases in cough (11.0% versus 6.4%), untreated diabetes (3.2% versus <1.0%), high blood pressure (13.1% versus 8.9%), cigarette smoking (12.7% versus 5.2%) and excessive alcohol consumption (5.8% versus 1.2%). Other conditions remained similar with 27.0% still disabled (6MWT<400m). 78 (13%) patients died or were lost-to-follow-up, with risk factors at end of treatment including on-going symptoms (RR1.7, 95%CI 1.1–2.7), high blood pressure (RR2.3, 95%CI 1.2–4.1) and undernutrition (RR2.6, 95%CI 1.7–3.9). Nine patients had recurrent TB. Employment status remained unchanged, with 47.5% still unemployed 18–24 months later.

**CONCLUSIONS:**

TB survivors experienced substantial multimorbidity 18–24 months post-TB treatment. Health services must integrate long-term care strategies to address these ongoing health challenges.

Despite reported treatment success rates exceeding 85% for people newly enrolled in first-line TB treatment, substantial numbers experience post-TB pulmonary or extra-pulmonary sequelae that reduce their quality of life.^1–3^ TB survivors also face significantly higher mortality than the general population.^4^ The overall disease burden is substantial with post-TB morbidity and mortality estimated to account for 58 million disability-adjusted life years (DALYS), representing 47% of the total TB DALY burden.^5^ With an estimated 155 million TB survivors globally, the need to improve post-TB health is imperative.^6^ Although there are clinical standards for assessing, managing and rehabilitating persons with post-TB lung disease,^7^ national TB programmes (NTP) have been slow to adopt these approaches. A few studies from China and several African countries have demonstrated the feasibility of assessing patients at the end of TB treatment for disability, comorbidities and risk factors within the routine health services.^8–10^ These studies show that those requiring further care can be identified and referred, though further research is needed to ensure those referred are receiving the care they require, and are contributing to the evidence base for WHO guidance on post-TB care.^11–12^

Addressing TB-associated disability and comorbidities up to two years after the end of TB treatment is part of national strategic plans in several countries.^13^ However, how this can be implemented within a routine NTP setting and its added value assessed remains unclear. We therefore conducted a study to follow-up patients in China, 18–24 months after completing TB treatment, to evaluate their health status and the on-going burden of symptoms, comorbidities, risk factors and disability. The overall aim was for the TB health facility staff in 11 selected health facilities in China to contact the 586 patients who successfully completed TB treatment between 2022–2023,^9^ and assess their status 18–24 months later.

Specific objectives were to: i) assess whether these follow-ups could be done and whether patient outcomes (alive, dead or lost to follow-up [LTFU]) could be determined; ii) evaluate the prevalence of symptoms, comorbidities, risk factors and disability (both new and pre-existing) and examine whether these conditions at the end of TB treatment were associated with later death or LTFU; iii) identify numbers diagnosed with recurrent TB; and iv) document employment status.

## METHODS

### Design

This was a cohort study involving primary data collection.

### Setting and study sites

The 11 health facilities that were selected for the previous China study were used for the current study.^9^ These sites were chosen based on their broad geographical coverage, sufficient numbers of registered TB patients, and staff willingness to attend to post-TB work without additional resources. All 11 facilities were equipped with on-site chest radiography, a laboratory for blood tests (including fasting blood glucose) and blood pressure measuring equipment.

### Study Population

The study population included 586 TB patients who successfully completed TB treatment in the previous project.^9^ The TB survivors were re-assessed between May–December 2024.

### Training health professionals involved in the study

Capacity building was done in accordance with the Structured Operational Research and Training Initiative (SORT IT) model.^14^ In brief, at project onset, a remote training module was implemented to refresh the Chinese focal points on key principles of operational research and data collection. They collaborated to finalize the generic protocol and agree on measurements to be done in each facility. This process was similarly applied after the study’s completion to analyse the data and prepare the paper.

### Assessment procedures using the structured evaluation form

As with the previous study, a focal point (the head or chief doctor) in each health facility was appointed to oversee and monitor TB survivor assessments 18–24 months post-TB treatment. Assessments were either carried out face-to-face in health facilities or conducted remotely by phone if TB survivors lived far away. Advice and referral were provided in case TB survivors needed further care. Assessments were done using the same assessment form as before,^9^ with the addition of employment status. The form was translated into Chinese, printed and sent to the selected health facilities for on-site evaluation. Data were collected on: TB registration number; demographics; clinical details; dates of end of TB treatment and 18-24 month reassessment; general health status (alive, died, LTFU); cause of death as reported by family, by healthcare authority or on the death certificate; symptoms; comorbidities (old and new), including diabetes and high blood pressure with measurements of fasting blood glucose and blood pressure – HBA1_C_ was not available; risk factors (old and new) including self-reported cigarette smoking and excess alcohol drinking, and undernutrition assessed by body mass index (BMI); recurrent TB, which was self-reported and verified in medical records; and employment status at the end of TB treatment and at the 18–24 month reassessment. Disability was assessed by performing the 6-minute walking test (6MWT) as previously described.^8,9^ For those undergoing remote assessments, individuals were guided in how to perform the 6MWT on a measured track. An abnormal 6MWT was defined as walking <400 meters in the 6 minutes.^8,9,15^

### Data variables, sources of data and data collection

Data variables were collected using the assessment forms between May–December 2024. Each focal point and data manager at the implementing sites cross-checked the data for accuracy. Data were entered into an Excel file and analysed using OpenEpi. Categorical data were presented as frequencies and proportions and continuous data as means and standard deviations (SD). Comparisons between groups were made using the chi square test. Conditions at the end of TB treatment associated with a combined outcome of death or LTFU (‘an adverse outcome’) during the 18–24 months post-TB treatment follow-up were analysed using the chi square test. Relative risks (RR) with 95% confidence intervals were used as measures of association. Significance levels were set at 5% (*P*<0.05).

The research protocol was approved by the Ethics Advisory Group of the Union (EAG 14/24, dated 28/03/2024) and the review committee at the Beijing Chest Hospital, Beijing, China (YNLX-2022-002, dated 16/02/2022). Written informed consent was obtained from individual patients using a consent form in Chinese.

## RESULTS

### Feasibility of assessments and general health status

Of the 586 individuals who successfully completed TB treatment, 503 (86%) were contacted and reassessed. Five were contacted but declined reassessment, 19 (3%) had died and 59 (10%) were LTFU. Among those reassessed, 170 (34%) were evaluated at the same hospital or clinic where they had completed TB treatment; 7 (1%) were assessed at a different hospital or clinic close to home; and 325 (65%) were assessed remotely at home by a doctor or nurse via telephone or video: their 6MWT was assessed using a measured track and their blood pressure and fasting blood glucose were measured at a nearby health facility. One individual was assessed at home by a doctor. Among the 19 who died, 3 succumbed to TB-related lung complications, 1 died from cardiovascular disease, 1 from cancer, 2 from other reasons, and 12 died from unknown reasons.

### Characteristics of those successfully reassessed

Of the 503 individuals reassessed, the mean age (SD) was 54.2±17.6 years. Their baseline characteristics at the time of TB diagnosis are shown in Table 1. The majority were male and resided in rural areas. Pulmonary disease was present in 94%, with more survivors having bacteriologically confirmed disease than clinically diagnosed disease (58% v 42%) and more with new compared with recurrent disease (91% v 9%). Ongoing symptoms, comorbidities, risk factors and disability at the time of reassessment compared with the end of TB treatment are shown in Table 2. Significant findings from those assessed 18–24 months later included an increased proportion with cough, with untreated diabetes, with high blood pressure, with current cigarette smoking and excess alcohol consumption. Other symptoms, comorbidities, risk factors and disability remained the same, with over a quarter of patients still classified as disabled at the 18–24 month assessment (6MWT<400m). Individuals who successfully completed treatment without symptoms, comorbidities, risk factors or disability but later developed new conditions are detailed in Table 3. In total, 16.2% of those with no symptoms at end of TB treatment became symptomatic, 13.5% developed a new comorbidity, 21.8% developed a new risk factor and 16.4% became disabled (6MWT<400m).

**Table 1.** Baseline characteristics (at the time of TB diagnosis) of individuals successfully reassessed 18–24 months after completing TB treatment in the eleven hospitals and clinics in China.

Baseline characteristics		Number (%)
Total		503
Gender:	Male	330 (65.6)
	Female	173 (34.4)
Residence:	Urban	191 (38.0)
	Rural	312 (62.0)
Disease location:	Pulmonary	472 (93.8)
	Extrapulmonary	31 (6.2)
Type of Tuberculosis:	Bacteriologically confirmed	292 (58.1)
	Clinically diagnosed	211 (41.9)
Category of Tuberculosis:	New	458 (91.0)
	Retreatment	45 (9.0)

**Table 2.** Symptoms, comorbidities, risk factors and disability in 503 individuals who successfully completed TB treatment and were reassessed 18–24 months later in China.

Category	Variable	End of TB treatment	18–24 months later
		Number (%)	Number (%)
Total cohort assessed		503	503
Symptomatology	No symptoms	419 (83.3)	416 (82.7)
	Any symptoms	84 (16.7)	87 (17.3)
	Cough	32 (6.4)	55 (11.0)*
	Shortness of breath	17 (3.4)	25 (5.0)
	Fatigue	30 (6.0)	25 (5.0)
	Chest pain	4 (<1.0)	11 (2.2)
	Other	25 (5.0)	8 (1.6)*
Comorbidities	Diabetes Mellitus on treatment^a^	57 (11.3)	58 (11.5)
	Diabetes Mellitus not on treatment^a^	3 (<1.0)	16 (3.2)*
	High blood pressure^b^	45 (8.9)	66 (13.1)*
	Mental Health Disorder	3 (<1.0)	6 (1.2)
Risk factors	Cigarette smoking	26 (5.2)	64 (12.7)**
	Excess alcohol drinking	6 (1.2)	29 (5.8)**
	Occupational exposure to silica dust	5 (1.0)	10 (2.0)
	Use of recreational drugs	0 (0)	0 (0)
	Undernutrition (BMI<18.5)	100 (19.9)	80 (15.9)
Disability	6MWT done	476 (94.6)	476 (94.6)
	6MWT <400 m	112 (23.5)	136 (27.0)^c^

a Includes individuals with previously diagnosed and newly diagnosed diabetes mellitus at the time of assessment;

b Includes individuals with previously diagnosed and newly diagnosed high blood pressure at the time of assessment;

c Denominator is the number undertaking 6MWT.

Comparisons of proportions by chi-square test:

* =P<0.05;

** =P<0.001

BMI = body mass index; 6MWT = 6-minute walking test.

**Table 3.** Individuals who completed TB treatment with NO symptoms, comorbidities, risk factors or disability and who developed one or more of these conditions when re-assessed 18–24 months later in China.

Characteristics		Number (%)
No symptoms at end of TB treatment		419
Symptoms at the 18–24 month re-assessment		68 (16.2)
	Cough^a^	46
	Shortness of breath	16
	Fatigue	17
	Chest Pain	7
	Other	4
No comorbidity at end of TB treatment		407
Comorbidities at the 18-24 month re-assessment		55 (13.5)
	Diabetes Mellitus^a^	17
	High Blood Pressure	41
	Mental Health Disorder	2
No risk factor at end of TB treatment		377
Risk factors at the 18–24 month re-assessment		82 (21.8)
	Cigarette smoking^a^	35
	Excess alcohol drinking	18
	Silica dust exposure	5
	Use of recreational drugs	0
	Undernutrition (BMI<18.5)	37
No disability at end of TB treatment	6MWT >400m	364
Tested for disability at 18–24 month re-assessment^b^	6MWT done	347
Disability at the 18–24 month re-assessment	6MWT <400m	57 (16.4)

a Numbers with specific symptoms, comorbidities and risk factors do not add up to the total as some patients had more than one of these health issues;

b 17 of the 364 patients who had no disability at end of TB treatment were unable to do the 6MWT at the reassessment.

BMI = body mass index; 6MWT = 6 minute walking test

### Characteristics at end of TB treatment of those who later died or were LTFU

Individuals who successfully completed TB treatment and had on-going symptoms, high blood pressure or undernutrition (BMI<18.5) at the end of treatment had a significantly higher risk of an adverse outcome (later death / LTFU) compared with those without these health issues (see Table 4).

**Table 4.** Symptoms, comorbidities, risk factors and disability in individuals successfully completing treatment who died or were lost-to-follow up 18–24 months later in China.

Category	Variable	End of TB Treatment	Died/LTFU 18–24 months later		RR	95% CI	P value
		Number	Number	(%)			
Total		586	78	(13.3)		
Symptomatology	Symptoms	105	21	(20.0)	1.7	(1.1–2.7)	<0.05
	No symptoms	481	57	(11.9)	ref		
Comorbidity	Diabetes	73	12	(16.4)	1.3	(0.7–2.2)	0.40
	No diabetes	513	66	(12.9)	ref		
	High blood pressure	32	9	(28.1)	2.3	(1.2–4.1)	<0.05
	No high blood pressure	554	69	(12.5)	ref		
	Mental health disorder	4	1	(25.0)	1.9	(0.3–10.5)	0.90
	No mental health disorder	582	77	(13.2)	ref		
Risk Factors	Cigarette smoking	27	1	(3.7)	0.3	(0.0–1.9)	0.20
	No cigarette smoking	559	77	(13.8)	ref		
	Excess alcohol drinking	6	0	-	1.2	(0.2–7.5)	1.0
	No alcohol drinking	580	78	(13.4)	ref		
	Silica dust exposure	5	0	-	1.4	(0.2–8.4)	1.0
	No silica dust exposure	581	78	(13.4)	ref		
	Undernutrition (BMI<18.5)	95	26	(27.5)	2.6	(1.7–3.9)	<0.001
	No undernutrition	491	52	(10.6)	ref		
Disability	6MWT done	557	76				
	6MWT<400m	137	24	(17.5)	1.4	(0.9–2.2)	0.12
	6MWT≥400m	420	52	(12.4)	ref		

Fisher Exact test applied when cell numbers <5.

LTFU = loss-to-follow-up; RR = relative risk; CI = confidence interval; BMI = body mass index; 6MWT = 6-minute walking test; ref = referent

### Recurrent TB

During follow-up, nine persons developed recurrent TB and were re-initiated on a new TB treatment regimen. Only one of the nine had a risk factor for TB (BMI<18.5) at the end of TB treatment.

### Employment status

There was no significant change in employment status between the end of TB treatment and 18–24 months later (see Table 5). Just under half of all patients were unemployed at the end of TB treatment and this remained unchanged.

**Table 5.** Employment status of the reassessed TB individuals 18–24 months after completing TB treatment in China.

Employment status	At the end of TB treatment	18–24 months later
	Number (%)	Number (%)
Total	503	503
Unemployed	236 (46.9)	239 (47.5)
Employed	96 (19.1)	93 (18.4)
Daily wage labourer	32 (6.4)	32 (6.4)
Homemaker	47 (9.3)	49 (9.7)
Student	10 (2.0)	8 (1.6)
Retired	58 (11.5)	63 (12.5)
Other	24 (4.8)	19 (3.8)

Unemployed defined as no paid work.

## DISCUSSION

This study marks the first attempt to contact and assess TB survivors 18–24 months after successfully completing treatment under routine NTP conditions in China. Several key findings emerged from this effort.

First, it proved challenging to contact the cohort as many individuals had dispersed throughout the country. For two thirds of survivors, the assessments had to be undertaken remotely at home via phone or video, which may mean that comorbidities or disability are not detected or are incorrectly assessed compared with face-to-face assessments. Nevertheless, this might be the only practical and affordable way to conduct such long-term follow-up within routine healthcare settings.

Second, it is concerning that 18–24 months after successfully completing TB treatment a significant proportion of TB survivors continued to experience on-going symptoms, comorbidities, risk factors and disability. Notably, some of these conditions (such as untreated diabetes, high blood pressure, cigarette smoking and excess alcohol consumption) had increased compared with levels observed at the end of TB treatment. Furthermore, between 10–25% of patients who were asymptomatic, without comorbidities, risk factors and disability at the end of TB treatment developed new symptoms or health issues during the subsequent follow-up period. This multimorbidity presents several risks. Smoking and excess alcohol consumption are well known risk factors for TB,^16,17^ with smoking also significantly associated with an increased risk of recurrent TB.^18,19^ Diabetes is associated with a reduced likelihood of recovering from TB, a higher risk of recurrent TB and an increased chance of developing multidrug-resistant TB.^20,21^ Untreated and poorly controlled diabetes, as was found in our cohort, further exacerbates the risk of TB.^22^ Smoking, excess alcohol intake, diabetes and high blood pressure are also important risk factors for non-communicable diseases, such as cardiovascular disease and cancer, and these may be responsible for some of the elevated all-cause mortality observed in TB survivors compared with the general population.^4^

Third, during the follow-up period, 78 (13%) patients died or were LTFU. Previous research investigating what happened to TB patients recorded as LTFU during treatment found that up to one third had died.^23^ Thus, it is possible that the actual number of deaths in our cohort is higher than reported. To account for this uncertainty, we combined death and LTFU as an adverse outcome and assessed whether there were any conditions at the end of TB treatment associated with it. We found that on-going symptoms, high blood pressure and undernutrition were all significantly associated with an increased risk of death/LTFU. This finding, although not surprising, is significant. On-going symptoms, particularly cough, might indicate undiagnosed chronic lung disease or lung cancer which, if untreated, are important causes of mortality.^24^ Additionally, high blood pressure in TB patients is associated with an increased mortality rate at 2 years of follow-up.^25^ Undernutrition is not only an added risk factor for TB,^26,27^ but also predicts long-term mortality over 2–3 years of follow-up.^28^ A recent modelling study suggested that nutritional interventions at the TB-affected household level could substantially avert TB-associated deaths.^29^

Fourth, nine individuals were recorded as having recurrent TB, although this number could be higher as some of the unknown deaths during follow-up might have been due to undiagnosed TB and some of those LTFU might have had TB. Nevertheless, the incidence rate of recorded recurrent TB over the follow-up period in our cohort is significantly higher than that estimated for new TB in China in 2022.^30^

Finally, and disappointingly, the employment status of TB survivors 18–24 months after completing TB treatment was unchanged, with a substantial proportion still unemployed. We are unable to compare these findings with other studies as we were unable to find relevant research on the subject, highlighting the need for in-depth analysis of this area.

This study had several strengths. Over 85% of individuals who had successfully completed TB treatment were contacted and reassessed, despite their dispersion around the country. We also used the same standardised assessment tools at the two time periods which strengthened the comparability of our findings. However, there were some limitations. Two thirds of TB survivors were assessed remotely, including conducting the 6MWT, raising concerns about the quality and accuracy of the assessments. We failed to collect information on the date or type of recurrent TB so were unable to plot incidence curves and estimate time to event. About 10% of the TB survivors were LTFU, leaving their true status unknown. Finally, we should have gathered information about employment status before the onset of TB to better understand the disease's impact on jobs and livelihoods. This is an area in need of further research.

## CONCLUSION

It is clear that this cohort of successfully treated TB patients in China experiences on-going multimorbidity and disability that significantly impacts their quality of life and reduces life expectancy. These findings underscore our previous argument that TB programmes should assess patients for these conditions at the start and end of TB treatment.^31^ After completing treatment, survivors must be educated about potential health issues so they can take responsibility for improving their situation. They also require referral to the general health services to ensure long-term management of high blood pressure and diabetes, support for quitting smoking and abstaining from excess alcohol consumption and further investigation for on-going cough. It is unrealistic to expect TB programmes to shoulder these long-term responsibilities alone. As the Lancet Commission on TB emphasized five years ago, we need fully integrated person- and family-centred services so that the multiple morbidities that pre-exist or arise during or after TB treatment are diagnosed and properly managed and treated.^32^ This aligns with Sustainable Development Goal 3 (SDG) that calls on countries to ‘ensure healthy lives and promote well-being at all ages’.^33^ To achieve this, countries must adopt Universal Health Coverage to address these interconnected health issues. Without this comprehensive and holistic response, the future will likely mirror the past and people with TB will continue to suffer the consequences.

## References

[1] Tadyanemhandu C, Int J Tuberc Lung Dis. 2020;24:657–658. 32718395 10.5588/ijtld.20.0143 PMC9172971

[2] Muñoz-Torrico M, Functional impact of sequelae in drug-susceptible and multidrug-resistant tuberculosis. Int J Tuberc Lung Dis. 2020;24:700–705. 32718403 10.5588/ijtld.19.0809

[3] Gupte AN, Assessment of lung function in successfully treated tuberculosis reveals high burden of ventilatory defects and COPD. PLoS One. 2019;14:e0217289. 31120971 10.1371/journal.pone.0217289 PMC6532904

[4] Romanowski K, Long-term all-cause mortality in people treated for tuberculosis: a systematic review and meta-analysis. Lancet Infect Dis. 2019;19:1129-1137. 31324519 10.1016/S1473-3099(19)30309-3

[5] Menzies NA, Lifetime burden of disease due to incident tuberculosis: a global reappraisal including post-tuberculosis sequelae. Lancet Glob Health. 2021;9:e1679-e1687. 34798027 10.1016/S2214-109X(21)00367-3 PMC8609280

[6] Chakaya J, Post-tuberculosis lung disease: is there a light at the end of the tunnel? Lancet Infect Dis 2024;24:677-679. 38527476 10.1016/S1473-3099(24)00136-1

[7] Migliori GB Clinical standards for the assessment, management and rehabilitation of post-TB lung disease. Int J Tuberc Lung Dis. 2021;25:797-813. 34615577 10.5588/ijtld.21.0425 PMC8504493

[8] Lin Y, Is it feasible to conduct post-tuberculosis assessments at the end of tuberculosis treatment under routine programmatic conditions in China? Trop Med Infect Dis. 2021;6:164. 34564548 10.3390/tropicalmed6030164 PMC8482211

[9] Liu Y, Managing comorbidities, factors and disability at start and end of TB treatment under routine program conditions in China. Trop Med Infect Dis. 2023;8:341. 37505637 10.3390/tropicalmed8070341 PMC10383887

[10] Adakun SA, on behalf of the Kenya, Uganda, Zambia and Zimbabwe TB Disability Study Group. Disability, comorbidities and risk factors at end of TB treatment in Kenya, Uganda, Zambia and Zimbabwe. IJTLD Open. 2024;1:197-205. 39022778 10.5588/ijtldopen.24.0082 PMC11249599

[11] World Health Organization. Policy brief on tuberculosis-associated disability. Geneva, Switzerland: WHO, 2023.

[12] Calvi M, Comprehensive care for people affected by TB: addressing TB-associated disabilities. Int J Tuberc Lung Dis 2024;1:195-196. 10.5588/ijtldopen.24.0167 PMC11249605 39022780

[13] Republic of Kenya. National Strategic Plan for Tuberculosis, Leprosy and Lung Health. 2023/2024 – 2027/2028. Executive Summary.

[14] Ramsay A, The Structured Operational Research and Training Initiative for public health programmes. Public Health Action 2014,4,79-84. 26399203 10.5588/pha.14.0011 PMC4539036

[15] Casanova C, The 6-min walk distance in healthy subjects: reference standards from seven countries. Eur Respir J. 2011;37:150–156. 20525717 10.1183/09031936.00194909

[16] Slama K, Tobacco and Tuberculosis: A qualitative systematic review and meta-analysis. Int J Tuberc Lung Dis 2007;11:1049-1061. 17945060

[17] Simou E, Alcohol consumption and risk of tuberculosis: A systematic review and meta-analysis. Int J Tuberc Lung Dis 2018;22:1277-1285. 30355406 10.5588/ijtld.18.0092

[18] d’Arc Lyra Batista J, Smoking increases the risk of relapse after successful TB treatment. Int J Epidemiol 2008;37:841-851. 18556729 10.1093/ije/dyn113 PMC2483312

[19] Lin Y, Tuberculosis recurrence over a 7-year follow-up period in successfully treated patients in a routine program setting in China: A prospective longitudinal study. Int J Infect Dis 2021;110:403-409. 34332089 10.1016/j.ijid.2021.07.057

[20] Baker MA, The impact of diabetes on tuberculosis treatment outcomes: A systematic review. BMC Med 2011;9:81. 21722362 10.1186/1741-7015-9-81 PMC3155828

[21] Liu Q, Diabetes mellitus and the risk of multidrug-resistant tuberculosis: A meta-analysis. Sci Rep 2017;7:1090. 28439071 10.1038/s41598-017-01213-5 PMC5430797

[22] Leung CC, Diabetic control and risk of tuberculosis: a cohort study. Am J Epidemiol 2008;167:1486-1494. 18400769 10.1093/aje/kwn075

[23] Kruyt ML, True status of smear-positive pulmonary tuberculosis in Malawi. Bull World Health Org 1999;77:386-391. 10361755 PMC2557676

[24] GBD 2021 Causes of Death Collaborators. Global burden of 288 causes of death and life expectancy decomposition in 204 countries and territories and 811 subnational locations. 1990-2021: a systematic analysis for the Global Burden of Disease Study 2021. Lancet 2024;403:2100-2132. 38582094 10.1016/S0140-6736(24)00367-2 PMC11126520

[25] Seegert AB, Hypertension is associated with increased mortality in patients with tuberculosis in Guinea-Bissau. Int J Infect Dis 2021;109:123-128. 34224869 10.1016/j.ijid.2021.06.062

[26] Cegielski J, The relationship between malnutrition and tuberculosis: Evidence. Int J Tuberc Lung Dis 2004;8:286-298. 15139466

[27] Lonnroth K, Consistent log-linear relationship between tuberculosis incidence and body mass index. Int J Epidemiol 2010;39:149-155. 19820104 10.1093/ije/dyp308

[28] Hu S, Nutritional indices predict all cause mortality in patients with multi-/rifampicin-drug resistant tuberculosis. Infect Drug Resist 2024;17:3253-3263. 39104459 10.2147/IDR.S457146 PMC11298562

[29] McQuaid CF, Estimating the epidemiological and economic impact of providing nutritional care for tuberculosis-affected households across India: a modelling study. Lancet Glob Health 2025;13(3):e381-e382 39824201 10.1016/S2214-109X(24)00505-9 PMC11865009

[30] World Health Organization. Global Tuberculosis Report 2024. WHO, Geneva, Switzerland. 2024.

[31] Harries AD, Why TB programmes should assess for comorbidities, factors and disability at the start and end of TB treatment. Int J Tuberc Lung Dis 2023;27:495-498. 37353872 10.5588/ijtld.23.0178

[32] Reid MJA, Building a tuberculosis-free world: the Lancet Commission on tuberculosis. Lancet 2019;393:1331-1384. 30904263 10.1016/S0140-6736(19)30024-8

[33] Sustainable Development Goals (website). New York, United Nations; 2022.

